# Supplementary material for: Dynamic spreading of chromatin-mediated gene silencing and reactivation between neighboring genes in single cells
Source: eLife. 2022 Jun 9;11:e75115. doi: 10.7554/eLife.75115 (PMC9183234; doi:10.7554/eLife.75115)

Figure 1 – figure supplement 2. Original gel images from RT-PCR.

B. K562 HDAC4 NS

Column 1 – Generuler 1kb + Ladder
Column 2 – run-on transcript 5 days -dox
Column 3 – run-on transcript 5 days +dox mCherry spike
Column 4 – run-on transcript 20 days -dox
Column 5 – run-on transcript 20 days +dox
Column 6 – run-on transcript no reverse transcriptase control
Column 7 – beta-actin control 5 days -dox
Column 8 – beta-actin control 5 days +dox mCherry spike
Column 9 – beta-actin control 20 days -dox
Column 10 – beta-actin control 20 days +dox
Column 11 – beta-actin no reverse transcriptase control
Column 12 – Generuler 1kb + Ladder

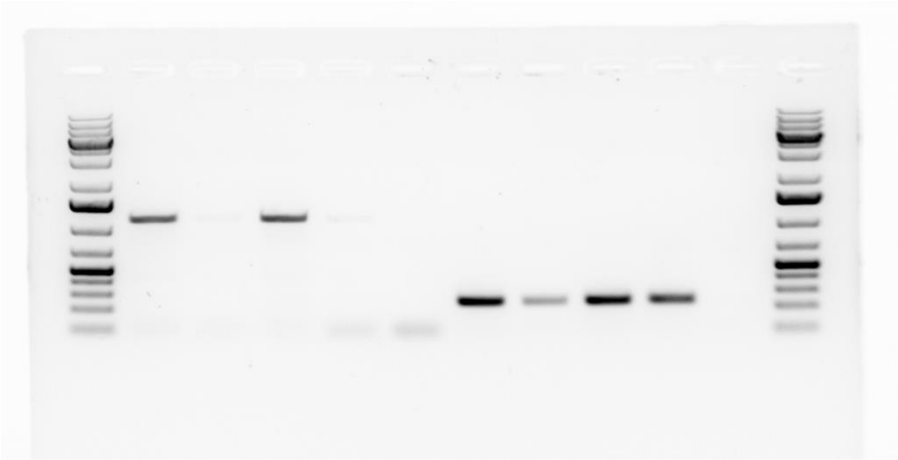

F. CHO HDAC4 SH

Run-on transcript (left gel in panel F)

Column 1 – Generuler 1kb + Ladder
Column 2 – clone 21 cDNA day 0
Column 3 – clone 21 cDNA day 1
Column 4 – clone 21 cDNA day 5
Column 5 – clone 21 no reverse transcriptase control
Column 6 – clone 21 gDNA
Column 7 – clone 26 cDNA day 0
Column 8 – clone 26 cDNA day 1
Column 9 – clone 26 cDNA day 5
Column 10 – clone 26 no reverse transcriptase control
Column 11 – clone 26 gDNA
Column 12 – Generuler 1kb + Ladder

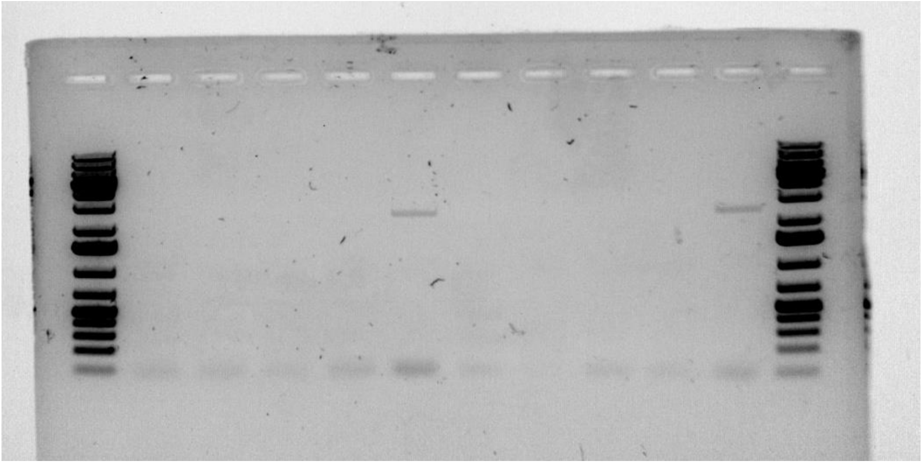

Beta-actin control (right gel in panel F)

Column 1 – Generuler 1kb + Ladder
Column 2 – clone 21 cDNA day 0
Column 3 – clone 21 cDNA day 1
Column 4 – clone 21 cDNA day 5
Column 5 – clone 21 no reverse transcriptase control
Column 6 – no sample
Column 7 – clone 26 cDNA day 0
Column 8 – clone 26 cDNA day 1
Column 9 – clone 26 cDNA day 5
Column 10 – clone 26 no reverse transcriptase control
Column 11 – no sample
Column 12 – Generuler 1kb + Ladder

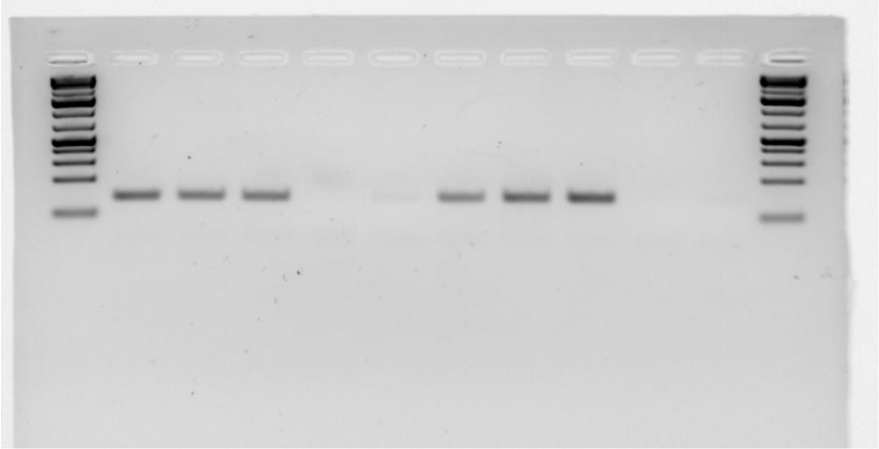

Supplement: Figure 1—figure supplement 4—source data 1. [file elife-75115-fig1-figsupp4-data1.zip › Figure 1 - figure supplement 4 - source data 1.pdf]
