# Supplementary figures and images for: Dynamic spreading of chromatin-mediated gene silencing and reactivation between neighboring genes in single cells
Source: eLife. 2022 Jun 9;11:e75115. doi: 10.7554/eLife.75115 (PMC9183234; doi:10.7554/eLife.75115)

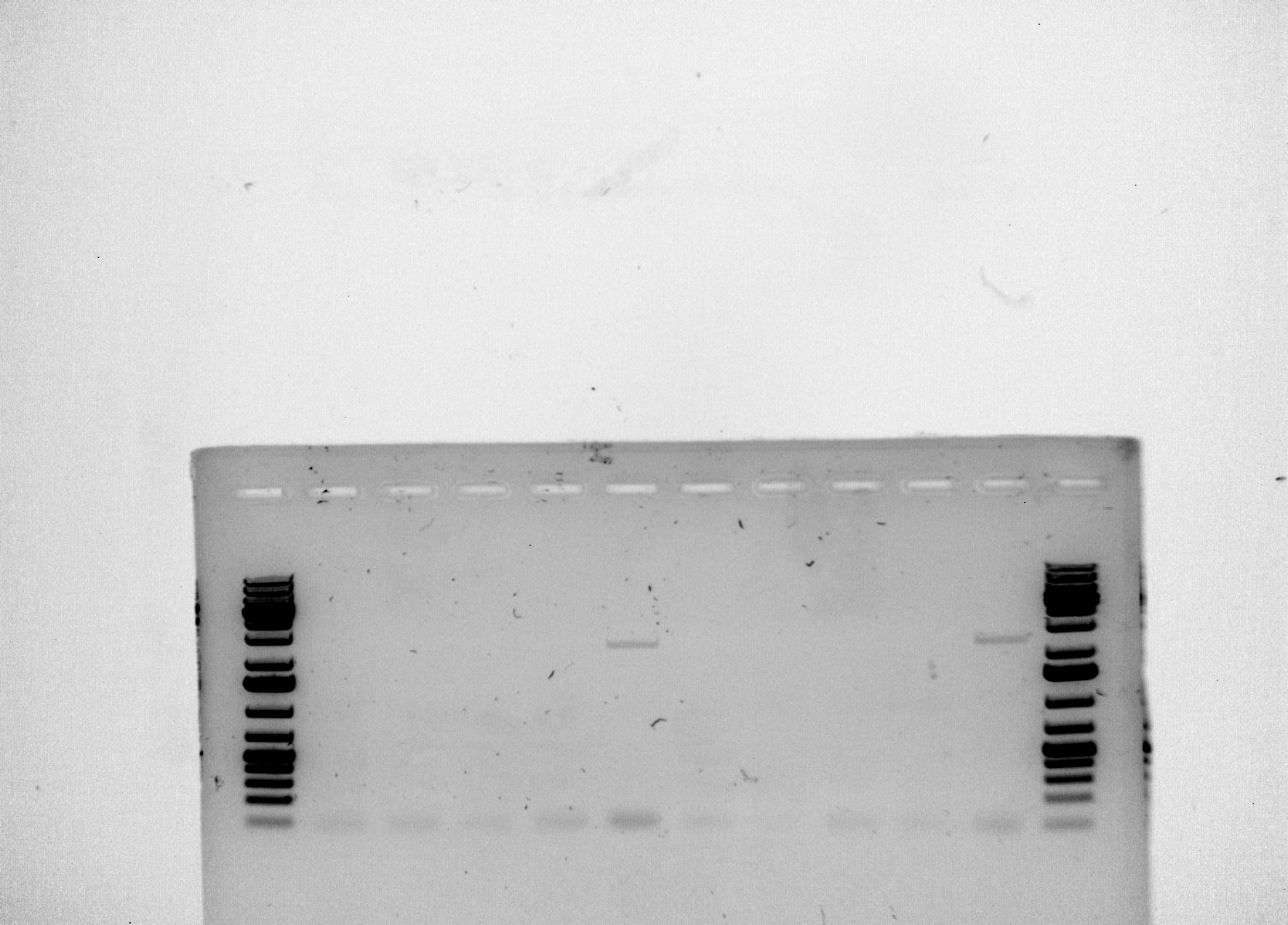

Supplement: Figure 1—figure supplement 4—source data 1. [file elife-75115-fig1-figsupp4-data1.zip › Figure 1 - figure supplement 4 - source data F_left.tif]

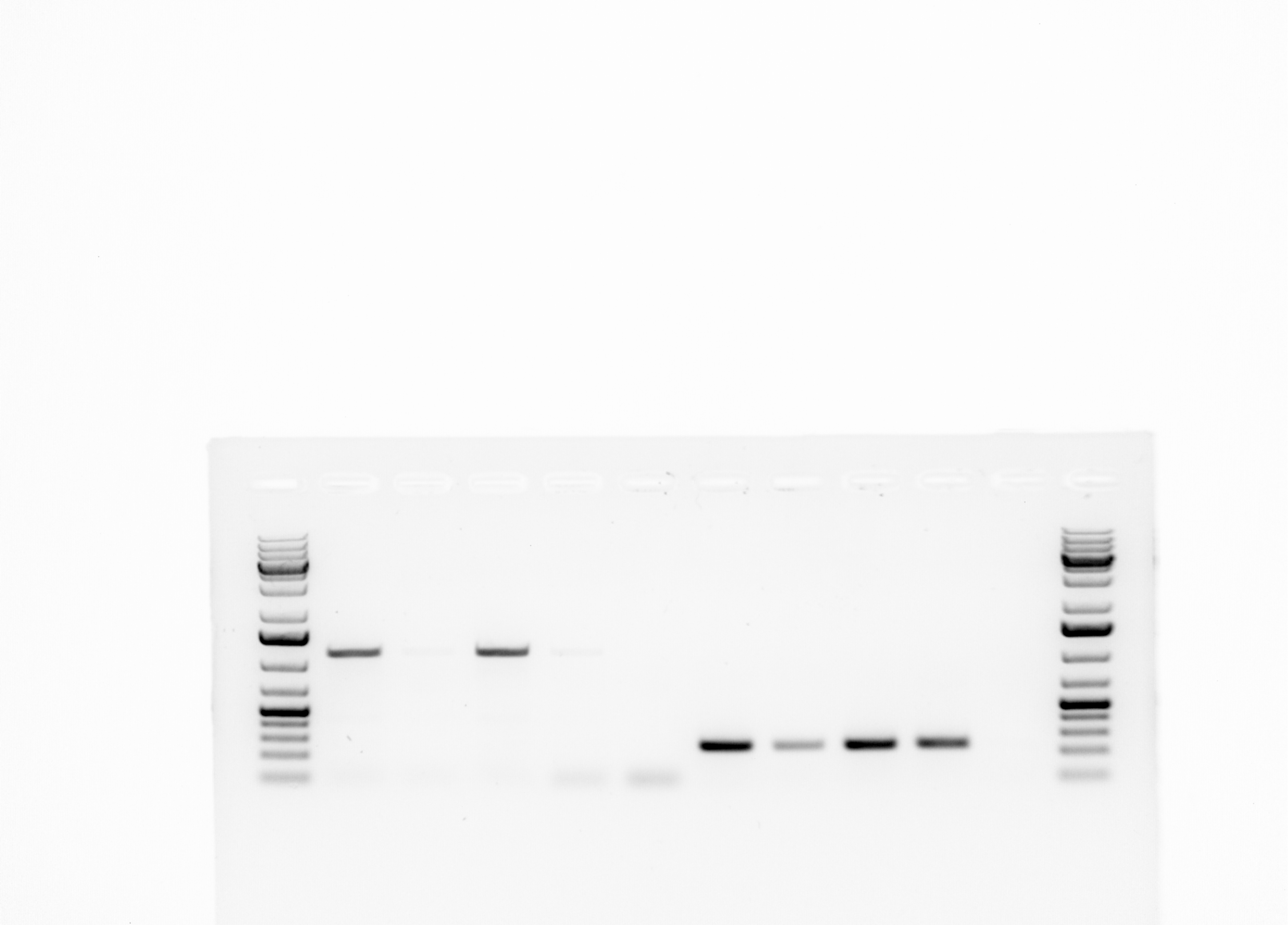

Supplement: Figure 1—figure supplement 4—source data 1. [file elife-75115-fig1-figsupp4-data1.zip › Figure 1 - figure supplement 4 - source data B.tif]

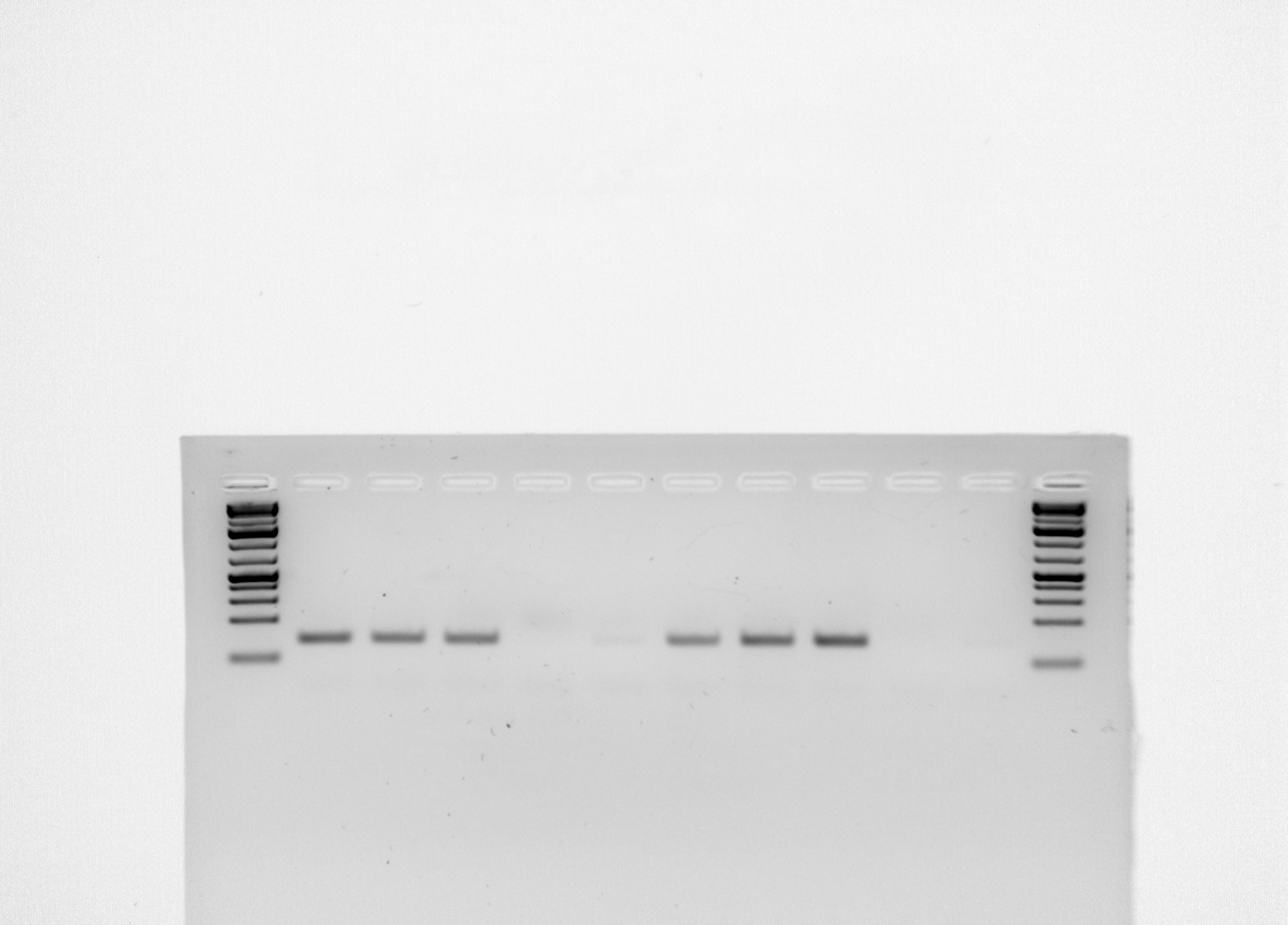

Supplement: Figure 1—figure supplement 4—source data 1. [file elife-75115-fig1-figsupp4-data1.zip › Figure 1 - figure supplement 4 - source data F_right.tif]
